# Efficacy of third-line therapy using bevacizumab in a patient with metastatic colorectal cancer

**DOI:** 10.3747/co.v16i5.395

**Published:** 2009-09

**Authors:** J. Gaulin, R. Kotb, E. Turcotte, G. Berard, B. Sawan, G. Schmutz, P. Beauregard

**Affiliations:** * Departments of Hematology and Oncology (Gaulin, Kotb, Beauregard), Nuclear Medicine (Turcotte), Clinical Pharmacy (Berard), Pathology (Sawan), Radiology (Schmutz), Centre Hospitalier Universitaire de Sherbrooke, Sherbrooke, QC

**Keywords:** Colorectal cancer, bevacizumab, third-line therapy

## Abstract

Bevacizumab is currently approved in association with first- and second-line 5-fluorouracil–based chemotherapy regimens for patients with metastatic colorectal cancer. Few data about the usefulness of bevacizumab in third-line settings are available. We describe a patient refractory to folfiri and folfox chemotherapy regimens who showed a dramatic and durable response to bevacizumab and folfiri. We also review and discuss the available literature.

## 1. INTRODUCTION

Colorectal cancer (crc) is the third most common malignancy affecting men and women in Canada 1. Many patients present with metastatic disease, and a significant proportion of those treated with cure-intended surgery, with or without adjuvant chemotherapy, eventually relapse 2.

Significant improvement in chemotherapy for metastatic colorectal cancer (mcrc) has been achieved with the addition of irinotecan and oxaliplatin to 5-fluorouracil (5fu)–based therapy 3–5. More recently, targeted agents such as bevacizumab, cetuximab, and panitumumab have shown promising results, especially in combination with conventional chemotherapy 6–13.

Bevacizumab is a human recombinant monoclonal antibody directed against the vascular endothelial growth factor. Combined with 5fu-based regimens, bevacizumab has been shown to improve outcome in the setting of first- and second-line chemotherapy 10–12, and it is now considered a standard of care for patients in those settings 12. Data concerning the usefulness of bevacizumab in the third-line treatment of mcrc are limited 14–17.

Here, we report on a patient diagnosed with mcrc who progressed after the usual folfiri and folfox regimens, but who achieved a significant tumour response with bevacizumab-containing third-line treatment.

## 2. CASE REPORT

A 49-year-old man with an unremarkable medical history was diagnosed with metastatic cecal adenocarcinoma in June 2005. The tumour was invading the right iliac fossa up to the superficial abdominal wall. This patient underwent extensive right hemicolectomy with partial abdominal wall resection on June 21. Pathology studies revealed a 7×8-cm infiltrative grade iii mucinous adenocarcinoma. Of the 36 lymph nodes collected, 7 were infiltrated, and some showed capsular rupture. Lymphatic vascular permeation and a peritumoural metastatic peritoneal implant were present. The right lateral resection margin was positive. Serum carcinoembryonic antigen (cea) dropped to 8.7 μg/l from 97.4 μg/l after surgery.

After a significant delay because of postoperative recovery and wound healing, combined positron-emission tomography (pet)–computed tomography (ct) and contrast-enhanced ct imaging in September 2005 in advance of chemotherapy showed a 15-mm hypermetabolic subcutaneous mass in the right axillary region and multiple metastatic lymph nodes and peritoneal implants. The patient received 12 cycles of folfiri, given every 2 weeks from October 2005 to March 2006. A combined pet–ct imaging study in april 2006 showed reduced metabolic activity, but stable disease according to the response Evaluation Criteria in Solid Tumors (recist) guidelines 18. A small chemotherapy pause was authorized; however, serum cea rapidly rose over the subsequent weeks, and pet–ct imaging in June 2006 demonstrated disease progression with 2 new intra-abdominal lesions, 1 along the psoas muscle and 1 along the right hepatic artery. Second-line chemotherapy with 8 cycles of folfox6 was given every 2 weeks from June to September 2006. Serum cea declined slightly after the first 3 cycles and then rose again under chemotherapy (to 16 μg/L from 23.5 μg/L, and then to 29 μg/L). A control pet–ct study in October 2006 demonstrated further disease progression of the right psoas muscle mass up to 4 cm.

For patient convenience, and also because of an early oxaliplatin-attributed sensory neuropathy, a switch was made to oral capecitabine 1250 mg/m^2^ twice daily for 14 days every 3 weeks. In March 2007, the pet–ct showed tumour progression, and serum cea was rising to 139 μg/l, but the patient was asymptomatic, and capecitabine was maintained. Control imaging in September 2007 revealed further enlargement of the known lesions and new psoas and peritoneal masses. The patient was experiencing right groin pain necessitating continuous opioid-based analgesia, together with limited right hip mobility. Serum cea continued to rise, reaching a high of 552 μg/l.

Capecitabine was stopped, and the patient received two courses of palliative radiotherapy (20 Gy in 5 fractions each time) in September and December 2007 for right groin and flank pain and reduced functional capacity.

**FIGURE 1:**
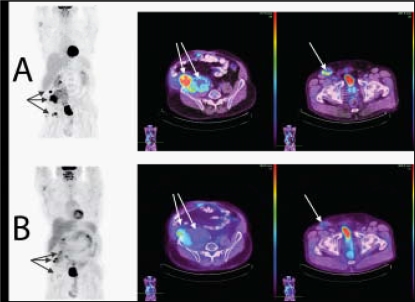
Combined positron-emission tomography and computed tomography images (A) before and (B) after third-line treatment.

In February 2008, awaiting bevacizumab approval, the patient received 2 cycles of folfiri, without significant clinical improvement. He then received 10 cycles of bevacizumab (5 mg/kg) every 2 weeks in addition to folfiri. Post-treatment evaluation showed near-normal functional capacity. The pain had resolved, and the patient was no longer taking analgesics. Serum cea dropped markedly to 24 μg/l, and new pet–ct imaging showed diminished metabolic activity and reduced sizes of most of the lymph nodes and metastatic implants, qualifying for a partial response according to recist criteria (Figure 1).

At November 2008, the patient was still on treatment, asymptomatic and in good general and functional status.

## 3. DISCUSSION AND CONCLUSIONS

Currently, seven chemotherapy drugs have been approved for patients with mcrc: 5fu, capecitabine, irinotecan, oxaliplatin, bevacizumab, cetuximab, and panitumumab. In the 1980s, 5fu was the only active drug for mcrc, and the addition of irinotecan and oxaliplatin improved response rate (rr), progression-free survival (pfs), and overall survival (os). A similar scenario is happening with targeted agents that are already showing benefits and promising results.

Bevacizumab was the first targeted agent to be approved for routine use in mcrc. Most clinical trials so far have evaluated bevacizumab in the first- and second-line settings, and data concerning its efficacy in third-line settings are scarce. The U.S. National Cancer Institute’s Treatment referral Center Trial by Chen *et al.* reported low rrs for third-line therapies using bevacizumab 14. Based on an assessment by the investigators, the rr was 4%, but an independent review evaluated the rate at 1%. The pfs was 3.5 months, and the os was 9 months. Another trial presented by Emmanouilides *et al.* concluded that bevacizumab could result in disease stabilization and clinical benefit in a proportion of heavily pretreated patients, with a median time to progression of 16 weeks 15. The use and benefits of bevacizumab in salvage therapy for pretreated patients with refractory crc are still unclear and must be assessed in randomized studies.

In June 2008, Shitara *et al.* reported on the efficacy and feasibility of bevacizumab-containing third-line chemotherapy in a patient with mcrc that had progressed under folfox and folfiri chemotherapy regimens 16. Similarly, a retrospective analysis of mcrc patients pretreated with folfiri and folfox and receiving salvage bevacizumab plus either folfiri or folfox demonstrated a rr of 9.5%. The median pfs and os were 5.3 months and 9.5 months respectively 17.

Our data accords with the three latter reports. Our patient initially presented stable disease and then rapid tumour progression after first-line folfiri chemotherapy, and despite further tumour progression under folfox and capecitabine chemotherapy, he presented spectacular clinical improvement, reduction of tumour markers, and partial response on ct scan under bevacizumab–folfiri third-line chemotherapy. All these data underline the potential role of bevacizumab-containing chemotherapy beyond second-line treatment, especially for patients who did not receive bevacizumab-containing first- or second-line chemotherapy. In such patients, the utility of bevacizumab-containing third-line therapy should be considered.
